# Cooperative and individualistic functions of the microRNAs in the miR-23a~27a~24-2 cluster and its implication in human diseases

**DOI:** 10.1186/1476-4598-9-232

**Published:** 2010-09-03

**Authors:** Ravindresh Chhabra, Richa Dubey, Neeru Saini

**Affiliations:** 1 Functional Genomics Unit, Institute of Genomics and Integrative Biology (CSIR), Mall Road, Delhi-110007, India

## Abstract

The small RNA molecules of about 19-22 nucleotides in length, aptly called microRNAs, perform the task of gene regulation in the cell. Interestingly, till the early nineties very little was known about them but eventually, the microRNAs have become forefront in the area of research. The huge number of microRNAs plus each one of them targeting a vast number of related as well as unrelated genes makes them very interesting molecules to study. To add to the mystery of miRNAs is the fact that the same miRNA can have antagonizing role in two different cell types i.e. in one cell type; the miRNA promotes proliferation whereas in another cell type the same miRNA inhibits proliferation. Another remarkable aspect of the microRNAs is that many of them exist in clusters. In humans alone, out of 721 microRNAs known, 247 of them occur in 64 clusters at an inter-miRNA distance of less than 5000bp. The reason for this clustering of miRNAs is not fully understood but since the miRNA clusters are evolutionary conserved, their significance cannot be ruled out. The objective of this review is to summarize the recent progress on the functional characterization of miR-23a~27a~24-2 cluster in humans in relation to various health and diseased conditions and to highlight the cooperative effects of the miRNAs of this cluster.

## Introduction

MicroRNAs (miRNAs) are non-coding RNAs which are initially transcribed from genomic DNA to long primary transcripts (pri-miRNAs) and then are cleaved by nuclear Drosha into 60-70 nucleotide hairpin-shaped precursor RNAs (pre-miRNAs) [1,2]. Pre-miRNAs are exported to the cytoplasm by Ran-GTP and Exportin-5 [3,4]. Once in the cytoplasm, the pre-miRNAs are cleaved by dicer into 22 nucleotide mature miRNA duplexes [5,6]. In association with RNA-induced silencing complex (RISC), one strand of the miRNA duplex binds to the target mRNA sequence in the 3'UTR (untranslated region). This binding finally leads to either the translational repression or degradation of the target mRNA.

The first micro RNA (lin-4) was found in *C. elegans *in 1993 [7] and since then thousands of miRNAs have been discovered in different species. In humans alone, 721 miRNAs have been identified till date [8]. Out of these, 247 miRNAs have been found to occur in 64 clusters at an inter-miRNA distance of less than 5000bp [8] and since most of these clusters show a higher degree of evolutionary conservation [9], it indicates that the clustering propensity of miRNAs might be mediating biological role. miRNAs are encoded in diverse genomic locations including intergenic regions, introns of protein-coding genes and introns/exons of noncoding RNA genes [8]. Literature suggests that a single miRNA can regulate many mRNA targets, and several miRNAs can regulate a single mRNA. They are involved in a variety of functions, including developmental transitions, neuronal patterning, apoptosis, adipogenesis metabolism and hematopoiesis in different organisms. Many independent studies have provided evidence for the possible association of miRNA(s) with several human malignancies and infectious diseases [10-12].

The miR-23a~27a~24-2 cluster is found to have altered expression in many diseased states (Table 1). This cluster has been shown to give rise to contrasting phenotypes in different cell types [13,14]. Several studies have also linked the expression of this cluster to cell cycle, proliferation, differentiation, haematopoesis and cardiac hypertrophy [13,15-17] to name a few, thereby indicating that miR-23a~27a~24-2 cluster controls several processes during health and diseases. Infact, this cluster was the first downstream miRNA target implicated in regulating the development of myeloid versus lymphoid cells [18]. It is well documented that all three miRNAs of this cluster are derived from a single primary transcript but depending on different biological conditions their expression pattern varies i.e. in some instances all three have similar expression pattern and in some one or two of these miRNA are expressed and the third is not (Table 1). Such complex expression pattern and association with diseased states makes it a very interesting subject for research. The objective of this review is to summarize the current knowledge about the miRNAs of miR-23a~27a~24-2 cluster in relation to various health and diseased conditions and to highlight the cooperative effects of the miRNAs of this cluster.

**Table 1:** The pathological conditions in which miRNAs of miR-23a~27a~24-2 cluster are implicated [102] (u - up, d - down, u/d - up in one study and down in another, NA - up/down is not known)

Diseased condition	23a	27a	24	23b	27b	Reference
Acute lymphoblastic leukemia (ALL)	u	u	u	u	u	[28]

Acute myeloid leukemia (AML)	u	u	u	u	u	[28]

Acute promyelocytic leukemia (APL)	d	d	d			[41]

Autism spectrum disorder (ASD)	d	d				[103]

Bladder cancer	u			u		[42]

Breast cancer		u				[15,50]

Cardiac hypertrophy	u	u	u	u	u	[17,104]

Colorectal cancer	d	d	u		d	[33,105]

Gastric cancer (stomach)	u	u	u			[32,33]

Glioblastoma	u		u	u		[106]

Heart failure	u		u	u		[107]

Hepatocellular carcinoma (HCC)	u	u	u			[13]

Kidney cancer		u				[108]

Lung cancer			u		d	[109]

Lupus nephritis						[110]

Malignant melanoma		d		d		[111]

Oral squamous cell carcinoma (OSCC)	d	d			d	[112]

Pancreatic cancer	u		u	u		[29,113]

Papillary thyroid carcinoma (PTC)			u			[114]

Prostate cancer	u/d	u/d	u	u/d	u/d	[115,116]

Schizophrenia			d			[117]

Serous ovarian cancer	u	u		u		[118]

Ulcerative colitis (UC)	u		u	u		[119]

Uterine leiomyoma (ULM)		u		u		[120]

Vesicular stomatitis			NA			[121]

### miR-23 cluster paralogs

**Figure 1:**
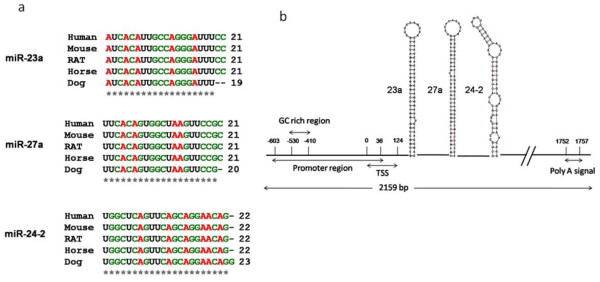
**Multispecies alignment and genomic organization of miR-23a~27a~24-2 cluster. a**. Alignment of the mature sequences of miR-23a, miR-27a and miR-24 among five species indicate that these miRNAs are highly conserved. **b**. The structure of the 2159 nucleotide pre-miR-transcript of hsa-mir-23a~27a~24-2, the structure of individual miRNAs shown is derived from the Mfold program [101]http://mfold.bioinfo.rpi.edu/cgi-bin/rna-form1.cgi with the input of their respective precursor sequences. The promoter region of this cluster spanning -603 to +36 is well characterized; it includes a GC rich region (-530 to -410) where transcription factors are predicted to bind and the transcription start site (TSS) spans 0 to 124 region. The poly-A tail is from 1752 to 1757.

Ancient gene duplications have given rise to miR-23 cluster paralogs in mammals: miR-23a~27a~24-2 cluster (localized on chromosome 9q22) is intergenic and its paralog miR-23b~27b~24-1 cluster (localized on chromosome 19p13) is intronic. It is not uncommon for miRNA clusters to have homologs and paralogs as shown by Yu et al in 2006 [19]. The miR-23a~27a~24-2 cluster encodes pri-miRNA transcript composed of 3 miRNAs: miR-23a, miR-27a and miR-24. Mature sequence of miR-23a and miR-27a differ by just one nucleotide in comparison to their paralogs miR-23b and miR-27b while the mature sequence of miR-24-1 and miR-24-2 is same. Since the mature sequence of paralogous miRNA is similar, one might speculate that they have overlapping targets. The miRNA target prediction software, TargetScan does predict common targets of miR-23a and miR-23b and miR-27a and miR-27b, and there are also reports where both the isoforms (27a & 27b or 23a & 23b) are simultaneously up regulated and down regulated (Table 1). The targets may be shared but since the paralogous clusters are present on different chromosomes and are produced from different transcripts, their regulation might be independent of each other. Hence, the functional differences between them would be because of differential regulation of their expression and processing rather than different portfolios of their potential targets [20]. Such duplication of miRNA cluster would lead to very rigid control of the targets of this cluster. But to decipher why such duplication occurred in the first place, one needs to study the evolution of this cluster. To understand the evolution of miR-23a and -23b clusters, we looked for their presence in different organisms (Fig. 1a, Additional File 1). We observed that these clusters are known to be present only in vertebrates and there are certain differences across species which are easily seen in the Additional File 1. However, very extensive study needs to be done to work out the evolution of these clusters. One such study has been done for miR-17 cluster which has 3 paralogous cluster, one each on chromosome 7, 13 and × by Tanzer et al in 2004 [21]. They have described DDC (duplication-degeneration-complementation) model for the evolution of miR-17 cluster which predicts that after a gene duplication either one of the two paralogs is lost or, if both are retained, then the two paralogs will evolve to perform complementary sub-functions. The model further predicts that duplicate clusters that still contain redundant miRNAs should differ in their spatio-temporal expression patterns. Extrapolating these findings on miR-23a and miR-23b cluster, one can say that herein both the paralogous clusters were retained that differ in their expression profiles and perform complementary functions.

The expression consistency of miRNAs in a miR-23 cluster has been shown in several studies (Table 1) but the cooperativity between them is yet to be worked out. Consistent with these studies, there is also a report which shows that miRNAs of a miRNA cluster might work in combination to accomplish their function [22]. Nevertheless, inconsistent expression of the miRNA members of miR-23 cluster has also been observed. Yu et al found miR-23a/23b cluster to be inconsistent while studying human miRNA clusters in six hematopoietic cell lines [19]. There was no detectable expression of miR-23b, miR-27b and miR-24 in all 6 cell lines (3D5, Jurkat, K562, CMK, HL-60, U937) but miR-23a and miR-27a was detected with similar expression levels in 4 cell lines (3D5, K562, HL-60, U937). This may be caused by their independent transcription or because they are transcribed together but processed by additional post transcriptional regulation mechanism.

### Regulation of the miRNA cluster

The primary transcript of miR-23a~27a~24-2 cluster is around 2.2 kb long [23]. It has been observed that miR-23a~27a~24-2 cluster gene lacks long open reading frame and the short open reading frame found does not code for any protein. Lee et al in 2004 showed for the first time that like protein-coding genes, the transcription of miR-23a~27a~24-2 cluster can be Pol II dependent [23]. Infact the region covering -603 and +36 bp contains the promoter of miR-23a~27a~24-2 cluster. It was also observed that the promoter of miR-23a~27a~24-2 cluster lacks both the known common as well as less common promoter elements such as TATA box, the initiator element, the downstream promoter element (DPE), or the TFIIB recognition element (BRE), downstream core element (DCE), and the MED-1 (multiple start site element downstream) [24]. It is not surprising as more than 80% of mammalian protein coding genes are driven by TATA less promoter. The nucleotide sequence of the promoter shows that the region covering -530 to -410 of the cluster is GC rich and contains at least two GC boxes and probably this is the site where transcription factor (s) binds [25]. They also observed that the miR-23a~27a~24-2 promoter is different from the promoters of RNA pol II transcribed snRNA genes as it lacks the proximal sequence element (PSE) too.

The schematic structure of miRNA cluster along with the promoter binding region has been shown in Fig. 1b.

Interestingly, even after the primary transcript of miRNA cluster is made, there is no guarantee that all three miRNAs will be formed proportionally. Previously, in a study done by our group we have observed increased expression of miR-27a as well as miR-24-2 but not of miR-23a after the over expression of miR-23a~27a~24-2 cluster in HEK293T cells [14]. In support to our findings there are reports in the literature. Buck et al also recently observed that down-regulation of miR-27a occurs independently from miR-23a and miR-24 [26]. In an independent study, Lee et al had previously observed a block in the processing of pri-miR-23a to mature miR-23a in HEK293 cells but not in HeLa cells and undifferentiated P19 cells [27]. Moreover inconsistent expression of the miRNA members of miR-23 cluster has also been observed. It seems that both transcriptional and post transcriptional mechanisms could be responsible for differential expression of miRNAs located within the same genomic cluster. All this indicates that the miRNAs of this cluster are under complex regulatory mechanisms which can act even after the primary transcript of miR-23a~27a~24-2 has been made.

### Functions of the miR-23a~27a~24-2 cluster

#### miR-23a~27a~24-2 cluster in tumorigenesis

Deregulation of microRNA(s) has been found in wide range of human diseases including cancer. However, it is not clear whether the observed change in the expression profile is a cause or consequence of pathological processes. Additionally, miRNA profiling studies have revealed that in various diseased conditions, all the three microRNAs of the miR-23a~27a~24-2 cluster are simultaneously deregulated (Table 1). Although, their significance is yet to be worked out in these conditions, but the simultaneous increase or decrease of mir-23a, miR-27a and miR-24-2 point towards their cooperative role in carrying out their functions in the diseased conditions. Expression of miR-23a~27a~24-2 cluster has been found to be up-regulated in acute lymphoblastic leukemia [28], acute myeloid leukemia [29], chronic lymphocytic leukemia [30], breast cancer [31], gastric cancer [32,33], cholangiocarcinoma cells [34] and hepatocellular carcinoma cells (HCC) [13] as compared to their respective normals. In accordance with the tumorigenic activity, Huang et al in 2008 found that over expression of miR-23a~27a~24-2 cluster could promote HCC cell growth and attenuate TGF-β-induced apoptotic cell death [13]. It has been observed that progressive tumors show high CDK2/4 activity, which could phosphorylate and inhibit the transcriptional activity of SMAD3, thus conferring cancer cells to escape from TGF-β - induced tumor suppression. Recently, Rogler et al in 2009 [35] has reported miR-23b cluster targets Smad 3, 4, and 5. The coordinate targeting of Smad3 mRNA by miR-23a cluster and miR-23b cluster adds an evolutionary advantage. Generally individual miRNAs have a weak role in knocking down gene expression [36,37] and an amplification scheme may be necessary to bring a complex and powerful signaling network such as TGF-β to a rapid halt [38]. In addition to the above, the role of miR-23a~27a~24-2 cluster has also been observed in metastasis and liver cirrhosis [13]. Cameron et al observed elevated expression of miR-23a, miR-24 and miR-27a in type III versus type I EBV (Epstein-Barr virus) latency [39]. The authors suggested that EBV-mediated regulation of cellular miRNAs may contribute to EBV signaling and might be associated with lymphoid and epithelial cancers. Table 1 lists the diseases where these miRNA have been implicated.

In contrast to the above cited studies on the widespread evidence of the increased expression of 23a~27a~24-2 cluster in various cancers, recently we observed that the over expression of miR-23a~27a~24-2 cluster in human embryonic kidney cells (HEK293T cells) induces apoptosis by caspase-dependent as well as caspase-independent pathway. Pro and anti-apoptotic nature of the miR-23a~27a~24-2 cluster suggest that this cluster may exhibit context-dependent functions and warrants urgent need to identify its molecular functions in humans, especially the downstream targets of this cluster.

### Function of the individual members of the miR-23a~27a~24-2 cluster

#### miR-23a

**Figure 2:**
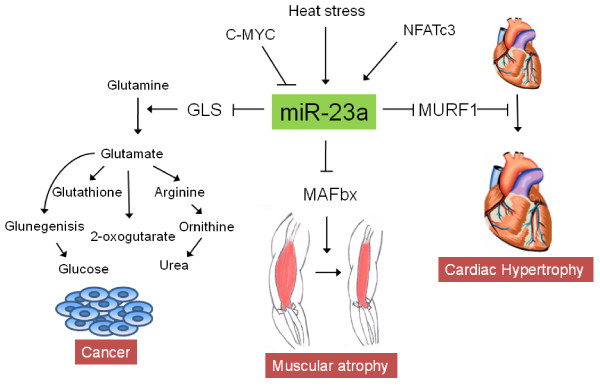
**Role of hsa-miR-23a in various pathological conditions**. During cancer, repression of miR-23a by MYC leads to up-regulation of glutaminase (GLS) thus mediating glutamine metabolism which is important for bioenergetics and maintaining redox homeostasis in cancer cells. The transcription factor NFATc3 induces miR-23a which down-regulates MURF1, an anti-hypertrophic protein leading to cardiac hypertrophy. Also, in the conditions of heat induced stress, the expression level of miR-23a increases and by down-regulating MAFbx (an atrophic factor), it provides resistance to muscle atrophy.

miR-23a is the first member of the mir-23a~27a~24-2 cluster. So far, miR-23a has been implicated in several cancers, cardiac hypertrophy and muscular atrophy (Fig. 2).

##### miR-23a in cancer

During cancer, oncogenic transcription factor c-Myc regulates the glutamine catabolism in addition to the regulation of cell cycle and glucose metabolism. Myc's role in stimulating glutamine catabolism has been, in part attributed through the repression of miR-23a and miR-23b. miR-23b originates from miR-23b~27b~24-1 cluster and has one nucleotide difference in the mature sequence in comparison to the mature sequence of miR-23a. Gao et al in 2009 observed that increase in c-Myc in human P-493 B lymphoma cells and PC3 prostate cancer cells leads to suppression of miR-23a/b which in turn results in greater expression of their target protein, mitochondrial glutaminase, which is required for bioenergetics, nucleotide biosynthesis and redox homeostasis in cancer cells [40]. Saumet et al in 2008 observed that miR-23a~27a~24-2 is directly repressed by the PML-RARA oncogene [41]. The PML-RARA oncogene is formed by the chromosomal translocation that fuses the gene encoding the retinoic acid receptor alpha (RARA) with the promyelocytic leukemia protein (PML) gene. This fused PML-RARA oncogene is associated with acute promyelocytic leukemia (APL) and is responsible for increasing the expression of key cancer proteins in APL.

On the contrary, there are a few reports wherein up-regulation of miR-23a has been observed in cancers. Significant up-regulation of miR-23a (and miR-27a) was reported by Meng et al in human bladder cancer [42] and by Gottardo et al in malignant cholangiocytes in comparison to their normal tissues [34]. Furthermore, Mi et al in his study showed that miR-23a along with miR-24 and miR-27a are also among several other miRNAs that have been differentially expressed between acute myeloid leukemia (AMLs) and acute lymphoblastic leukemia (ALLs) [28]. Apart from these, up-regulation of miR-23a/b along with miR-24 has also been reported in osteoblast cell line [43].

Although the increase/decrease of miR-23a has been reported in most of the studies related to cancer but it is as yet unknown whether it is a cause or effect of the disease. Apart from playing a role in cancer metabolism, selective expression of miR-23a along with miR-24 has been shown in microvascular endothelial cells in vivo [44]. Also, miR-23a may play a role in angiogenesis as it is among the highly expressed miRNAs in human umbilical vein endothelial cells (HUVECs) [45]. It seems that a re-evaluation of cancer metabolism considering glutamine catabolism along with selective expression of miRNAs in the vasculature is needed before cancer can be effectively targeted in therapy.

##### miR-23a in cardiac hypertrophy

Cardiac hypertrophy is thickening of heart muscle resulting in decrease in size of the ventricles. It occurs in response to stress conditions like hypertension, the heart muscle damage and is an early indication of most of the heart diseases [46,47]. Lin et al in their study reported that miR-23a is a pro-hypertrophic miRNA, and its expression is regulated by the transcription factor, nuclear factor of activated T cells (NFATc3) [17]. They found that NFATc3 could directly activate miR-23a expression through the transcriptional machinery. Interestingly the muscle specific ring finger protein 1 (MuRF1), an anti-hypertrophic protein, has been identified as a target of miR-23a. It seems miR-23a conveys the hypertrophic signal by suppressing the translation of MuRF1. It was also observed that it is only miR-23a and not miR-27a and miR-24 which participate in initiating hypertrophy induced by hypertrophic stimuli including isoproterenol and aldosterone. The up-regulation of the other two miRNAs clustered with miR-23a, miR-27a and miR-24 occurred much later. It was suggested by the authors that since miR-23a is located closer to the transcription start site (TSS), the effect must be reaching the miR-23a first and later to other miRNAs.

##### miR-23a in muscle atrophy

Skeletal muscle atrophy is observed in many physiological and pathological settings including fasting, several chronic diseases like cancer, diabetes, AIDS, sepsis, and sarcopenia. During atrophy, atrophic factors up-regulate the muscle-specific F-box protein MAFbx/atrogin-1. Wada et al in 2008 observed that miR-23a plays anti-atrophic effect. It has been observed that miR-23a suppresses MAFbx/atrogin-1 translation by binding to 3' UTR of the mRNA and forced expression of miR-23a in myotubes and myofibers results in resistance to muscle atrophy [48].

The control of genes with antagonizing functions [cardiac hypertrophy (increased cell growth) and muscle hypertrophy (reduced cell growth)] may underlie the complex and conflicting effects of miR-23a.

#### miR-27a

**Figure 3:**
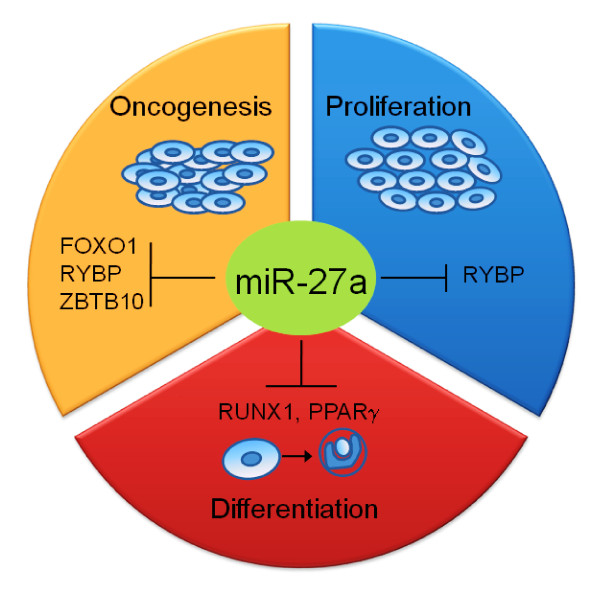
**Importance of hsa-miR-27a in oncogenesis, proliferation and differentiation**. miR-27a induces oncogenesis by negatively regulating FOXO1 (transcription factor that regulates genes involved in apoptosis and cell cycle progression), RYBP (pro-apoptotic gene) and ZBTB10 (repressor of Sp proteins which are known to cause proliferative and angiogenic phenotype in cancer cells), mediates differentiation by down regulating RUNX1 (regulator of haematopoesis) and PPARγ and also promotes proliferation by targeting RYBP (pro-apoptotic gene).

miR-27a is the microRNA sandwiched between the miR-23a and miR-24-2. Its paralogue, miR-27b originates from miR-23b~27b~24-1 cluster. Some of the cellular targets of miR-27a are those that can impact cell cycle regulation, proliferation, apoptosis and differentiation (Fig. 3)

##### miR-27a in cancer and multidrug resistance of cancer

Oncogenic role of miR-27a is confirmed by several experimental studies. miR-27a is significantly up-regulated in renal cell carcinoma [42], in cervical cancer [49], in gastric adenocarcinoma [32] and in breast cancer [15,50]. miR-27a is also involved in Helicobacter pylori induced infection and gastric cancer [51]. It has been found that in gastric cancer cells, miR-27a promotes the tumor development by targeting a tumor suppressor prohibitin, an evolutionary conserved and ubiquitous protein interacting with pRb and its family members [32]. In breast cancer cells, miR-27a functions as an oncogene by indirectly enhancing the expression of specificity protein (Sp) transcription factors. Sp factors have also been found to be over expressed in tumours where they contribute to the proliferative and angiogenic phenotype associated with cancer cells by regulating number of angiogenic proteins like vascular endothelial growth factor (VEGF), VEGF receptor 1 (VEGFR1, Flt-1), VEGFR2 (KDR) and the antiapoptotic gene survivin [52-55]. miR-27a down-regulates the repressor of the Sp proteins, ZBTB10/RINZF (a putative zinc finger and BTB domain containing protein) in both MDA-MB-231 breast cancer cells [15] and SKBr3 breast cancer cells [56].

miR-27a also contributes to oncogenesis by regulating cell cycle progression. It was observed that inhibition of miR-27a increased the percentage of cells in G2-M phase. This arrest was attributed to the induction of Myt-1 [15], which is responsible for phosphorylation of CDC2 resulting in inhibition of CDC2/cyclin B dependent initiation of mitosis [57-59]. The study by Chintharlapalli et al in 2009 showed that repression of miR-27a leading to the induction of ZBTB10 and Myt-1 was the cause behind apoptotic and anti-carcinogenic activity of Methyl 2-cyano-3,11-dioxo-18β-olean-1,12-dien-30-oate (CDODAMe) in colon cancer cells [60].

Guttilla et al had shown in 2009 that miR-27a along with miR-96, and miR-182 down-regulates FOXO-1 protein, a transcription factor that orchestrates the regulation of genes involved in the apoptotic response, cell cycle checkpoints, and cellular metabolism [50]. These three microRNAs, miR-27a, miR-96 and miR-182, were observed to be highly expressed in MCF-7 breast cancer cells, in which the level of FOXO1 protein is very low. It was also observed that over expression of FOXO1 resulted in decreased cell viability because of inhibition of cell cycle traverse and induction of cell death. It seems that targeting of FOXO1 by microRNAs may contribute to transformation or maintenance of an oncogenic state in breast cancer cells.

Apart from suppressing ZBTB10/RINZF and FOXO-1, miR-27a contributes to oncogenesis by negatively regulating RYBP/DEDAF, an apoptotic facilitator as has been reported in SKBr3 cells [56].

In addition to the oncogenic activity, miR-27a also regulates multidrug resistance. In two separate studies, it was shown that the down-regulation of miR-27a leads to decrease in the expression of multidrug resistance gene (MDR1/P-glycoprotein/ABCB1) [61,62]. Down-regulation of miR-27a also caused a decrease in Bcl-2 expression and increase in the Bax expression, thereby leading the cancerous cells towards apoptosis [62]. Also, Sun et al showed that genistein significantly inhibited growth of uveal melanoma cells and affects miR-27a and target gene expression [63]. Very recently van Jaarsveld et al has found significant association between miR-27a and chemotherapy resistance in ovarian cancer [64].

Furthermore while studying the effect of SNPs in miRNAs, Yang et al in their study found that the SNP rs895819, located in the terminal loop of pre-miRNA-27a, showed a protective effect in a large familial breast cancer study [65]. They also show that artificial mutations in the terminal loop of miR-27a can block the maturation process of the miRNA. They hypothesized that the G-variant of rs895819 might impair the maturation of the oncogenic miR-27a and thus, is associated with familial breast cancer risk. In an independent study, significant association between miR-27a polymorphism and gastric mucosal atrophy has been observed in Japanese male subjects [66].

Interestingly, Eitan et al in 2009 observed high expression of hsa-mir-27a in a sub-group of patients with very poor prognosis [67].

##### miR-27a in osteoarthritis pathological process

The effect of miR-27a on overall regulation of matrix metalloprotease-13 (MMP-13) and insulin-like growth factor binding protein (IGFBP), two genes involved in osteoarthritis (OA) pathophysiology adds another level of complexity to miR-27a mediated gene regulation. Tardif et al in their study showed that miR-27a indirectly decreases both matrix metalloprotease-13 (MMP-13) and the insulin-like growth factor binding protein (IGFBP) [68]. Since MMP-13 degrades a wide range of matrix components and IGFBP-5 plays an important storage role for anabolic factor IGF-1, this regulation by miR-27a could open up novel avenues in OA therapeutic strategies.

In addition to the regulatory role of miR-27a in diverse processes, miR-27a has also been associated with viral infections. It has been observed that both host and virus contain mechanisms to regulate miRNA expression and/or activity [26].

##### miR-27a in differentiation

miR-27a has been identified as a negative regulator of adipocyte differentiation in a study by Kim et al in 2010 [69]. It has been observed that the expression of miR-27a decreases during adipogenesis while expression of peroxisome proliferators-activated receptor-gamma (PPARγ) increases during adipogenesis. Lin et al in an independent study showed that miR-27a block the transcriptional induction of PPARγ and C/EBPα [70]. Since PPARγ has been implicated in the pathology of numerous diseases (including obesity, diabetes, atherosclerosis and cancer) and miR-27a has been found to suppress PPARγ; it appears that miR-27a could be a novel therapeutic target against metabolic complications associated with obesity as well as cancer.

In another study, Ben-Ami et al observed that miR-27a attenuates the expression of transcription factor RUNX1, a key regulator of hematopoiesis. In K562 cells, expression of RUNX1, a lineage-specific gene expression regulator has been found to increase during megakaryocyte differentiation. It seems that there exists a feedback loop mechanism between miR-27a and RUNX1 during megakaryopoiesis as the RUNX1 binding sites have been found upstream of miR-27a and the increase in miR-27a expression has been concomitantly found with the binding of RUNX1 to miR-27a [71].

miRNA 27a has also been found to play a critical role during osteogenesis. Schoolmeesters et al in their study in 2009 observed that grancalcin (GCA), a regulator of osteogenesis in human mesenchymal stem cells (hMSC) is a target of miR-27a [72]. It was also observed that expression of miR-27a decreases upon osteogeneic differentiation.

##### miR-27a regulates fat metabolism and cell proliferation

Ji et al in 2009 observed that miR-27a along with miR-27b were up-regulated in activated Hepatic stellate cells (HSCs). Under normal conditions HSCs reside in the space of Disse, containing bunches of vitamin A-riching lipid droplets and upon activatation HSCs lose cytoplasmic lipid droplets and trans-differentiate to proliferative, fibrogenic myofibroblasts, and play an essential role in the formation of liver fibrosis. It was also observed by the authors that down-regulation of over-expressed miR-27a and 27b allowed activated HSCs to accumulate cytoplasmic lipid droplets leading to decreased HSCs proliferation. They also observed that the fatty acid metabolism and cell proliferation regulating properties of miR-27a and miR-27b maybe, at least partly mediated by affecting RXRα expression [73]. It has been documented that RXRα plays a central role in adipogenesis, probably as a heterodimeric partner for peroxisome proliferator-activated receptor. RXRα also suppresses DNA synthesis and causes cell growth arrest in a variety of cell types including HSCs.

#### miR-24

miR-24-2 is unique among miRNAs of this cluster since miR-24 can originate from two different genomic loci one localized on chromosome 19p13 encoding miR-23b, -27b, and -24-1, and the other localized on chromosome 9q22 encoding miR-23a, -27a, and -24-2.

**Figure 4:**
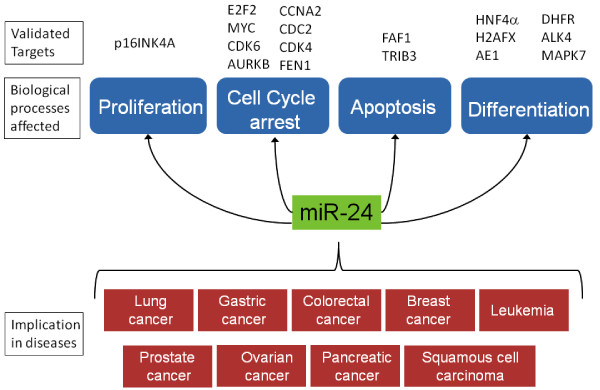
**Involvement of hsa-miR-24 in biological processes and the diseased states**. The different biological processes and the diseased states where the role of hsa-miR-24 has been established are shown. The experimentally validated target genes of miR-24 are depicted along with the respective biological processes.

Since, mir-24-1 and miR-24-2 have the same mature sequence but different primary transcripts, it means that they have similar biological functions but both of them are differentially expressed and regulated. A number of targets of miR-24 have been validated and its role in various diseases have been studied (Fig. 4).

##### miR-24 in haematopoetic differentiation

miR-24 is among many miRNAs which have been found to play a profound role in haematopoetic differentiation. Wang et al in 2008 reported that miR-24 targets human activin type I receptor ALK4 (hALK4) and modulates erythropoeisis. Activin in cooperation with erythropoietin plays an important role in differentiation of erythroid progenitors. Activin binds to type II activin receptor, leading to the recruitment, phosphorylation, and activation of type I activin receptor (ALK4, also known as ActRIB). The activated ALK4 phosphorylates SMAD2 and SMAD3, which, upon phosphorylation, form a heterocomplex with Co-SMAD (SMAD4), and the resulting SMAD complex binds to the promoter of the target genes, and regulates their expression. The authors show that miR-24 down-regulated hALK4 and attenuated the transcriptional responses of activin [74].

In a separate study, Lal et al in 2009 observed that miR-24 regulates the histone variant H2AFX, a key double-stranded break (DSB) repair protein during post mitotic differentiation of hematopoietic cell lines [16]. The reduction in miR-24 levels correlate with enhanced H2AFX mRNA and protein levels. Increased miR-24 levels have been found during differentiation while diminished miR-24 levels have been found in primary human blood cells. Moreover, it has also been observed that miR-24-mediated suppression of H2AFX renders cells hypersensitive to gamma-irradiation and genotoxic drugs.

##### miR-24 in tumorigenesis

Both the miR-24 loci (9q and 19p) are shown to be frequently altered in oral squamous cell carcinoma (OSCC). Recently, Lin et al (2010) found that miR-24 was up-regulated in OSCC tissues, plasma and OSCC cell lines in comparison to normals. The authors also observed that miR-24 might be contributing to the growth of OSCC cells by targeting p57(KIP2), a member of the KIP (kinase inhibitory protein) family of CKI, a potential tumor suppressor gene [75].

Several reports in the literature show that miR-24 might function differently in different cells. miR-24 promotes proliferation of transforming growth factor β-treated hepatocellular carcinoma cells (HuH7) as well as A549 lung carcinoma cells and inhibits proliferation in HeLa cells [76]. miR-24 promotes proliferation by indirect suppression of CHEK1 (which participates in G2-M checkpoint), BRCA1 (that activates double-strand break repair), CDKN1B (cyclin D inhibitor, p27Kip1) and VHL (tumor suppressor gene). Previous study by Lal et al further strengthened the proliferative function of miR-24 where they have shown that miR-24 directly suppresses CDK inhibitor p16INK4A which arrests cells in the G1 phase [77]. It is well known that over expression of Fas-associated factor-1 (FAF-1) stimulates apoptosis and down-regulation contributes to tumorogenesis. Qin et al showed that miR-24 can be a potential target for gene and drug therapy to treat hormone insensitive prostate cancer cells as they observed that increase of FAF-1 after miR-24 down-regulation induced significant apoptosis in DU-145, HGC-27, MGC-803 and HeLa cells [78]. Also, the anti-apoptotic role played by miR-24 might aid in cellular proliferation.

Anti-proliferative activity of miR-24 can be attributed through the regulation of multiple targets of cell cycle. Lal et al showed that miR-24 suppresses several key genes that regulate cell-cycle progression like MYC and E2F2 and several genes downstream of E2F2 and MYC i.e., AURKB, CCNA2, CDK4, CDC2 and FEN1 [79]. Additionally, they have also shown that MCM4, MCM10, RRM2 and PCNA are indirectly down-regulated by miR-24. Moreover it was observed that in addition to HepG2 and K562 cells this anti-proliferative effect can also be seen in human diploid fibroblasts.

Another level of miR-24 regulation in cell cycle progression came to light when Takagi et al found that miR-24 down-regulates the expression of HNF4α, a transcription factor regulating endo/xenobiotic-metabolism [80]. The down-regulation of HNF4α by miR-24 resulted in the decrease of various target genes such as cytochrome P450 7A1 and 8B1 as well as decrease of the S phase population in HepG2 cells. They also observed that the levels of miR-24 increase in HepG2 cells when treated with PMA, a protein kinase C/mitogen-activated protein kinase (PKC/MAPK) activator and H_2_O_2_, reactive oxygen species (ROS) generator. Since activation of PKC pathway induces cholestasis [81] and ROS pathway has a role in steato-hepatitis [82] and in both the diseases HNF4α is down-regulated [83-85], they inferred that this could be because of induction of miR-24.

Another role of miR-24 where they contribute to tumorigenesis was shown by Mishra et al. They showed that miR-24 regulates expression of the dihydrofolate reductase (DHFR), a key enzyme in intracellular folate metabolism and target of methotrexate (MTX), an important chemotherapeutic agent widely used in the treatment of several malignancies [86]. It was observed that a miR-24 target site polymorphism in DHFR 3' UTR results in loss of miR-24-function and high DHFR levels in the cell imparts a growth advantage to immortalized cells and induces neoplastic transformation. Enough evidence is there to say that miR-24 is deregulated in human colorectal cancer tumors and a subset of tumors has reduced levels of miR-24. These results are consistent with the data reported by Wu et al [87]. Their experiments indicated reduced expression of miR-24 in gastric cancer cells. In their study they also observed that miR-24 is a key factor for the induction of anion exchanger-1 (AE1). Functional AE1 is an erythroid-specific integral membrane protein expressed on the surface of mature erythrocyte. Under normal conditions full length AE1 is silenced at protein level in other tissues and has been found to impact the differentiation of erythroid lineage cells. AE1 mediates the Cl^-^/HCO3^- ^exchange across the plasma membrane, regulates intracellular pH and interacts with ankyrin, protein 4.1, spectrin which comprise the erythrocyte cytoskeleton. Increased expression of AE1 has been found in the cytoplasm in gastric and adenocarcinoma. AE1 interacts with another mir-24 target tumor suppressor p16 and sequesters p16 in the cytoplasm, thereby leading to loss of control of cell-cycle regulation and induction of gastric carcinogenesis. Transfection of a miR-24 into gastric cancer cells reduced the elevation of the AE1 protein, which resulted in return of AE1-sequestrated p16 to the nucleus, thereby inhibiting proliferation of the cells. The undetectable expression of AE1 in K562 cells suggested that miR-24 may take part in the tumorigenesis of erythroleukemia by arresting erythroid maturation through silencing of AE1 expression.

mir-24 emerged as a biomarker specific for Kaposi Sarcoma (KS) in a study done by O'Hara et al in 2009 to identify specific miRNAs that serve as biomarkers for tumor progression [88]. Apart from differentiation, proliferation and cell cycle regulation, miR-24 has also been found to be among the miRNAs which are Hodgkin lymphoma (HL) specific [89]. Recently, Liu et al in 2010 identified it as the differentially expressed miRNA involved in HCV entry, replication and propagation [90].

##### miR-24 in phenotypic plasticity

Phenotypic plasticity is essential for vascular development and vascular remodeling after injury as well as vascular proliferative diseases like atherosclerosis, pulmonary arterial hypertension etc. In response to injury, vascular smooth muscle cells (vSMCs) undergo phenotypic modulation from quiescent contractile phenotype to a proliferative synthetic state. Transforming growth factor-β (TGFβ) and Bone morphogenetic protein (BMP) pathways stimulate contractile phenotype whereas PDGF (platelet-derived growth factor)-signaling pathway promotes synthetic pathway. Recently Chan et al in 2010 observed that miR-24 is a key regulator of the crosstalk between the pro-contractile TGFβ/BMP signal and the pro-synthetic PDGF signal. The authors observed that hat PDGF-BB induces miR-24, which in turn leads to down-regulation of Tribbles-like protein-3 (TRIB3) [91]. Repression of TRIB3 coincides with reduced expression of SMAD proteins and decrease in BMP and TGFβ signaling, promoting a synthetic (proliferative) phenotype in vascular smooth-muscle-cell (vSMC) rather than quiescent (contractile) phenotype. Inhibition of miR-24 by antisense oligonuclotides abrogates the down-regulation of TRIB3 as well as pro-synthetic activity of the PDGF-signaling pathway.

### Biological relevance of miR-23a~27a~24-2 cluster as revealed by bioinformatic analysis

The overall cellular functions and pathways affected by co-expressed miRNAs remains largely undiscovered due to lack of high throughput target validation methods and such studies mostly rely on computational analysis of potential mRNA targets. Hence, most of the research on miRNAs to date has focused on individualistic effects of a miRNA. However, there are still a few reports which suggest that the miRNAs in a miRNA cluster effectively cooperate by targeting the individual genes of the same pathway thus justifying their coexistence [22,92]. To reveal biological significance of miR-23a~27a~24-2 cluster, a list of predicted targets of miR-23a, miR-27a and miR-24 was made using the TargetScan program, miRNA target prediction software (Additional File 2) [93,94]. From this list, we discovered that although the three miRNAs of this cluster shared very few predicted mRNA targets (Table 2) but still all the three miRNAs were reported either up-regulated or down-regulated simultaneously in several pathological states (Table 1); thereby indicating a possible connection between their target mRNAs.

**Table 2:** The common genes among the predicted target genes of miRNAs of miR-23a~27a~24-2 cluster in TargetScan

Gene Name	Definition
ADAM19	ADAM metallopeptidase domain 19 (meltrin beta)

ADAMTS6	ADAM metallopeptidase with thrombospondin type 1 motif, 6

CSNK1G1	Casein kinase 1, gamma 1

EBF3	Early B-cell factor 3

KPNA3	Karyopherin alpha 3 (importin alpha 4)

LIMK2	LIM domain kinase 2

NCOA1	Nuclear receptor coactivator 1

NLK	Nemo-like kinase

PLEKHH2	Pleckstrin homology domain containing, family H (with MyTH4 domain) member 2

RAP1B	RAP1B, member of RAS oncogene family

SOCS6	Suppressor of cytokine signaling 6

**Figure 5:**
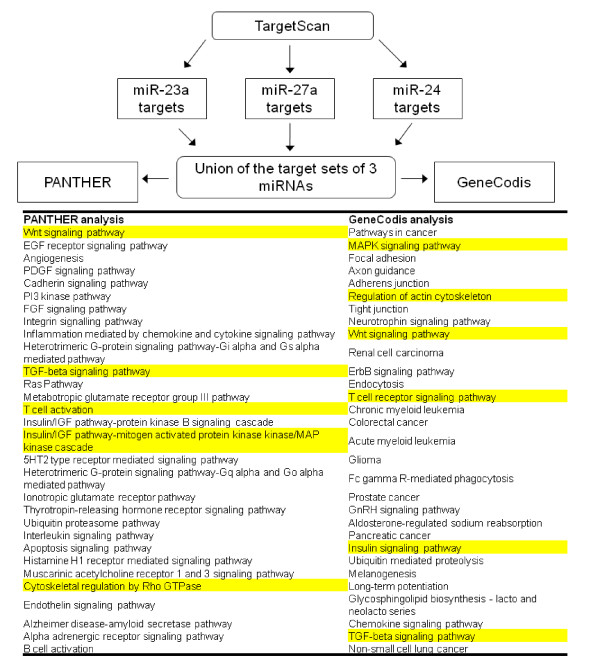
**Workflow for the bioinformatics analysis of predicted targets of miR-23a, -27a and -24**. Overview of the bioinformatics analysis of the TargetScan predicted targets of hsa-miR-23a, hsa-miR-27a and hsa-miR-24. The list of the pathways which crossed the p-value < 0.01 in PANTHER analysis is given in Additional File 3 and which crossed the p-value < 0.01 in GeneCodis analysis is given in Additional File 4. The pathways highlighted in yellow came out to be common among the enriched pathways of both PANTHER and GeneCodis analysis.

To evaluate the specific pathways or processes that are targeted by the miRNAs of this cluster, we used the list from TargetScan program to find enriched pathways by GeneCodis [95,96] and PANTHER analysis [97]. The PANTHER analysis revealed many signaling pathways as the enriched categories (Fig. 5, Additional File 3). In GeneCodis analysis, in addition to signaling pathways, several diseases viz., colorectal cancer, pancreatic cancer, renal carcinoma, chronic myeloid leukemia, acute myeloid leukemia and glioma are among the statistically significant enriched categories (Fig. 5, Additional File 4). It is noteworthy that the differential expression of miR-23a, miR-24-2 and miR-27a has already been reported in many of these diseased states (Table 1).

**Figure 6:**
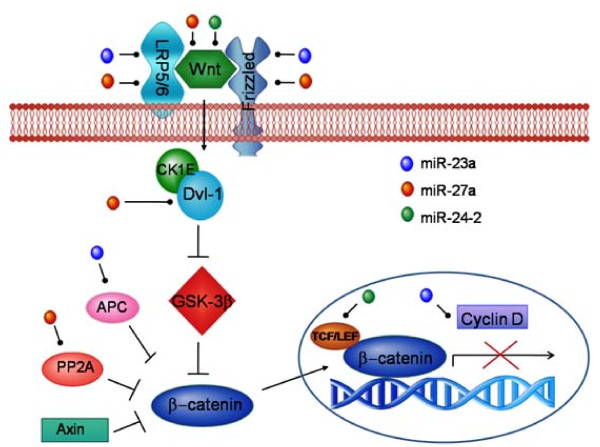
**Speculated role of miR-23a~27a~24-2 cluster in regulation of Wnt Signaling**. In normal circumstances, β-catenin protein is under the inhibitory effect of GSK-3β which leads to its degradation but when the Wnt signaling proteins bind to its receptors Frizzled and LRP-5 and -6; it leads to the activation of disheveled protein. This protein inhibits GSK-3β, thus preventing the degradation of β-catenin. Once the β-catenin is stabilized, it translocates to the nucleus and regulates the gene expression. In the various pathological conditions where this cluster is over expressed as is the case in many cancerous tissues, the Wnt genes; WNT3A and WNT4 could be targeted by miR-27a and miR-24 respectively, LRP5 and LRP6 by miR-23a and miR-27a respectively and FZD4 and FZD7 are targeted by both miR-23a and miR-27a. This leads to the inactivation of DVL1 protein. Also, DVL1 could itself be down-regulated by miR-27a. As a result, GSK-3β causes degradation of β-catenin, thus preventing its translocation to the nucleus and ultimately the transcription. To conserve the cells energy resources β-catenin associated genes like Cyclin D (targeted by miR-23a) and TCF7 (targeted by miR-24) are also inhibited. Among the other inhibitors of β-catenin (APC, Axin and PP2A), APC is targeted by miR-23a and PP2A by miR-27a, maybe these inhibitors are not required once GSK-3β is there to degrade β-catenin.

Interestingly, we observed that the Wnt, MAPK, TGF-β pathways, Insulin signaling, T cell receptor signaling and cytoskeletal regulation were the enriched categories (p-value < 0.01) in both PANTHER and GeneCodis analysis. There are a few reports which have talked about the involvement of miRNAs of this cluster in both MAPK signaling [98,99] and TGF-β signaling [13,91,100]. Since we got Wnt signaling as the enriched pathway in the similar bioinformatic analysis for all three miRNAs separately also (data not shown); we tried to illustrate the cooperativity of miRNAs of this cluster in regulating Wnt signaling (Fig. 6).

Wnt-β-catenin signaling controls critical events in development, and its dysregulation leads to cancers and developmental disorders. In normal circumstances, β-catenin protein is under the inhibitory effect of GSK-3β which leads to its degradation but when the Wnt signaling proteins bind to its receptors Frizzled and LRP-5 and -6; it leads to the activation of disheveled protein. This protein is an inhibitor of GSK-3β, thus preventing the degradation of β-catenin. The stable β-catenin moves to the nucleus and regulates the gene expression. As shown in Fig. 6 the various genes involved in Wnt signaling pathway may be targeted by the miRNAs of the miR-23a~27a~24-2 cluster. If that is the case then this cluster would lead to degradation of β-catenin resulting in blockage of transcription. It is remarkable that a few genes of the Wnt pathway are targeted by more than one miRNA thus indicating strong association among miRNAs and their stringent regulation of the pathway.

This in-silico analysis implies that the miRNAs present in this cluster could complement each other in regulating a certain pathway. Although, the targets used for this study are bioinformatically predicted and not validated, Further characterization of the functional interaction between Wnt signaling/β-catenin and miRNAs of miR-23a~27a~24-2 cluster will be required to clarify the role of miR-23a~27a~24-2 cluster in regulating Wnt signaling.

In many studies, individual effect of a miRNA may appear to be small but when they cooperate, the effect can be of significant proportions. The cooperativity among miRNAs is thus an interesting area of study and can change our perception of how we look at the miRNA mediated gene regulation.

## Conclusions

miRNAs are complex molecules and since they target a host of genes, their functions in a cell type are highly dependent on the repertoire of the genes present in that cell. In one type of cell, a miRNA can act as an apoptotic factor and in another the same miRNA can act as proliferation promoting factor. The fact that the miRNAs can be under the control of other genes further adds to their complexity. For instance, Kong et al recently reported that the miR-23a~27a~24-2 transcript is a downstream target of PU.1 and this cluster is the causal factor behind PU.1 promoting the myeloid differentiation over lymphoid differentiation [18]. As a result, it is highly improbable to characterize the functions of miRNAs and annotate them as yet. So far, most of the research in miRNA has been focused on the role of individual miRNA on regulating a particular gene. But, it is quite imperative to study the cooperative effects of miRNAs on regulating a cellular pathway or deciding the fate of a cell. This insight will give us a holistic picture of the miRNA regulation in the cell. The miRNAs present together in a cluster seems the most obvious subject to decipher the cooperative effects of miRNAs. There are a few reports in the literature which talk about cooperation among miRNAs in clusters. Yuan et al. have investigated the extent of coordination of miRNAs in regulating protein-protein interaction network [22]. They have shown that clustered miRNAs jointly regulate proteins which lie in close proximity in the interaction network. Kim et al have added a new dimension to this by reporting that separate clusters can also be co-expressed and have related activities [92]. Herein, we have discussed the individual functions of miRNAs of miR-23a~27a~24-2 cluster that have been experimentally validated and looked at their role in cooperation with each other. For studying the cooperative effects of miRNA in the cluster, we obtained the list of predicted targets of miR-23a, miR-27a, and miR-24 from TargetScan and used them for discovering enriched pathways. The analysis revealed the various enriched pathways and diseases, thus justifying their occurrence together in a single transcript and under the control of a single promoter. Although, this bioinformatic analysis needs to be validated experimentally, such a study would go a long way in the understanding of miRNA clusters. The study of miRNAs in coordination with each other is a must if we want to exploit miRNAs for therapeutic purposes.

## Competing interests

The authors declare that they have no competing interests.

## Authors' contributions

RC and NS conceived the study. The survey of the literature and the inferences were made by RC, RD and NS. The bioinformatics analysis was carried out by RC. The manuscript was drafted by all the authors. All the authors have read and approved the final manuscript.

## Supplementary Material

Additional file 1 **Presence/Absence of miRNAs of the two paralogous clusters in vertebrates**. The presence/absence of miRNAs (-23a, 27a, -24, -23b, -27b) in vertebrates as indicated in miRBase is represented in a table. Click here for file

Additional file 2 **Predicted targets of hsa-miR-23a, hsa-miR-27a and hsa-miR-24 by TargetScan**. This file contains the list of targets of hsa-miR-23a, hsa-miR-27a and hsa-miR-24 as predicted by the TargetScan. The union of the targets of these three miRNAs is used for bioinformatic analysis as reported in the manuscript. Click here for file

Additional file 3 **Enriched pathways from PANTHER analysis**. This file contains the list of enriched pathways obtained using PANTHER analysis that satisfies the criteria of p-value < 0.01. Click here for file

Additional file 4 **Enriched pathways from GeneCodis analysis**. This file contains the list of enriched pathways obtained using GeneCodis analysis that satisfies the criteria of p-value < 0.01. Click here for file
